# Profiling microRNA expression in Arabidopsis pollen using microRNA array and real-time PCR

**DOI:** 10.1186/1471-2229-9-87

**Published:** 2009-07-10

**Authors:** Carrie Chambers, Bin Shuai

**Affiliations:** 1 Department of Biological Sciences, Wichita State University, Wichita, KS 67260, USA

## Abstract

**Background:**

MicroRNAs (miRNAs) are ~22-nt small non-coding RNAs that regulate the expression of specific target genes in many eukaryotes. In higher plants, miRNAs are involved in developmental processes and stress responses. Sexual reproduction in flowering plants relies on pollen, the male gametophyte, to deliver sperm cells to fertilize the egg cell hidden in the embryo sac. Studies indicated that post-transcriptional processes are important for regulating gene expression during pollen function. However, we still have very limited knowledge on the involved gene regulatory mechanisms. Especially, the function of miRNAs in pollen remains unknown.

**Results:**

Using miRCURY LNA array technology, we have profiled the expression of 70 known miRNAs (representing 121 miRBase IDs) in Arabidopsis mature pollen, and compared the expression of these miRNAs in pollen and young inflorescence. Thirty-seven probes on the array were identified using RNAs isolated from mature pollen, 26 of which showed significant differences in expression between mature pollen and inflorescence. Real-time PCR based on TaqMan miRNA assays confirmed the expression of 22 miRNAs in mature pollen, and identified 8 additional miRNAs that were expressed at low level in mature pollen. However, the expression of 11 miRNA that were identified on the array could not be confirmed by the Taqman miRNA assays. Analyses of transcriptome data for some miRNA target genes indicated that miRNAs are functional in pollen.

**Conclusion:**

In summary, our results showed that some known miRNAs were expressed in Arabidopsis mature pollen, with most of them being low abundant. The results can be utilized in future research to study post-transcriptional gene regulation in pollen function.

## Background

MicroRNAs (miRNAs) are ~22-nt noncoding RNAs processed from their precursors by RNase III enzyme Dicer, which digests the hairpin structure in the precursor into miRNA:miRNA* duplexes. One strand in the duplex becomes mature miRNA that is incorporated with protein factors to form RNA-induced silencing complexes (RISCs) [1]. MiRNAs subsequently guide the RISCs to target mRNA molecules, where they silence the expression of the cognate genes by mRNA cleavage via the endoribonuclease activity of Argonaute (AGO) protein or by translation repression [2,3].

Cloning and bioinformatics approaches have identified many miRNAs in different eukaryotic species [4,5]. Large scale sequencing approaches have been employed to explore small RNAs at the genome level [6]. Up to date, there are 9539 miRNA entries in the miRBase (Release 13.0, March 2009) , which includes 187 miRNAs identified and confirmed in Arabidopsis [7]. Arabidopsis miRNAs function as regulators in a diverse range of processes including root, leaf and flower development, stress response, pathogen responses and mineral nutrient homeostasis [8-12].

Pollen plays the male role during sexual reproduction in higher plants, therefore, how pollen develops and functions has been intensively studied (reviewed in [13-15]). The pollen grain, also referred to as the male gametophyte, is a three-celled organism that is developed in the anther from pollen mother cells (PMC) through meiosis and two rounds of mitosis. Each PMC undergoes meiosis to form four microspores in a tetrad that is enclosed in a thick callose wall. The microspores are freed from the thick wall by the action of callase, an enzyme secreted from the tapetum layer of the anther, and become free uninucleate microspores. Development of microspores into pollen requires two mitotic divisions. The first mitosis is asymmetric and produces bicellular pollen that consists of a large vegetative cell and a small generative cell. The generative cell in bicellular pollen undergoes the second mitosis to form the two sperm cells. The timing of the second mitosis varies in different plant families. In Arabidopsis, the second mitosis occurs within the anther and produces a tricellular pollen grain. In most plant species, mature pollen is released from the anther in a partially dehydrated state. When it lands on the stigma, the pollen grain hydrates and the pollen tube grows out from the vegetative cell. The pollen tube extends through the transmitting tract of the style by tip growth and delivers the two sperm cells to the embryo sac to achieve double fertilization [14]

The function of pollen during germination, tube elongation and interaction with the female component relies on the proper regulation of gene expression. It is believed that the transcripts required for these processes have been produced and stored in mature pollen, and protein synthesis rather than transcription is the key factor controlling the production of the required products [16,17]. Transcriptome studies have identified thousands of genes expressed in different developmental stages of the male gametophyte [17,18]. Proteomic analysis has also been conducted to identify the functional products in mature pollen [19]. However, our knowledge on the regulatory link between the transcripts and protein products is very limited. We have no information on whether important regulators like miRNAs play any role in pollen function. To fill this knowledge gap, we have conducted a large scale analysis of miRNA expression in Arabidopsis mature pollen using miRNA array and real-time PCR techniques. Our results indicated that ~60% of known Arabidopsis miRNAs are expressed in mature pollen, and most of them are present at lower levels when compared with those in young inflorescence tissue. Our results also point out the sensitivity and reproducibility of the two different techniques. Based on our data, we question the effectiveness of using the array technology for analyzing miRNA expression.

## Results and discussion

### Genes in RNA silencing pathway are expressed in mature pollen

**Figure 1:**
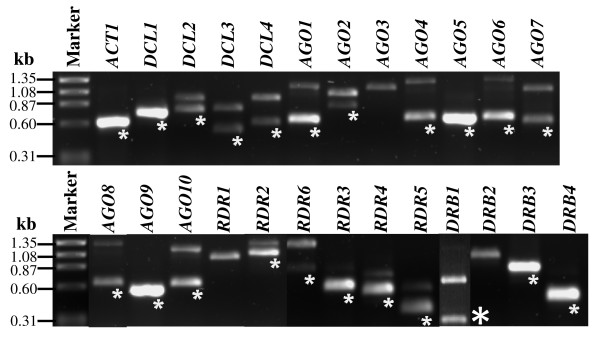
**Expression of RNA silencing pathway genes in Arabidopsis mature pollen**. *ACT1*(*ACTIN1*) was used as a positive control to ensure the quality of RNA and cDNA. PCR products amplified from cDNA are indicated by asterisks. Sequences for the cDNAs were confirmed by cloning and sequencing. PCR products at higher molecular weight in each sample were amplified from genomic DNA. *DCL: Dicer-like; AGO, Argonaut; RDR, RNA-dependent RNA polymerases; DRB, Double-stranded RNA binding protein. AGO3*, *RDR1 *and *DRB2 *were not detected.

Our lack of knowledge of miRNA functioning in mature pollen may be explained by inactivation of the RNA silencing pathway or by functional redundancy. A pollen transcriptome study by Pina et. al. (2005) suggested the first possibility in Arabidopsis mature pollen. They have shown that 15 genes in the RNA silencing pathway, including members of *DCL (Dicer-like)*, *AGO*, *RNA-dependent RNA polymerases *(*RDR) *families, were absent in mature pollen [18]. To examine this possibility, reverse transcriptase PCR (RT-PCR) was used to inspect the expression of all members of the *DCL*, *AGO*, *RDR *and *Double-stranded RNA binding protein *(*DRB*) genes (Figure 1). The results indicated that most of the genes in RNA silencing pathways were expressed in mature pollen, especially genes required for miRNA biogenesis and function such as *DCL1*, *AGO1 *and *DRB1/HYL1*. One possible explanation for the discrepancy in these results could be due to the different methods used to isolate pollen. Pina et. al. [18] used Fluorescence-Activated Cell Sorting (FACS) to isolate pollen grains to eliminate tissue contamination, whereas samples used in this study was isolated using a modified hand vacuum device [20]. To rule out the possibility of RNA impurity due to tissue contamination, *ACTIN7 *was used as a negative control in RT-PCR. *ACTIN7 *is strongly expressed in vegetative tissues, but not expressed in pollen [21]. No product was detected for *ACTIN7 *in our RNA samples using the same number of cycles in PCR reactions as for other genes (see Additional file 1), indicating that the observed positive results were not due to the tissue contamination. Results from RT-PCR in this study and microarray data from two transcriptome studies were further compared (see Additional files 2). We conclude that the differences between these results were mainly due to the limitation of the microarray technology. Problems associated with sensitivity and reproducibility have been reported for microarray analyses, even for the ones conducted with manufactured Affymetrix Gene Chips [22]. A comparison on the expression of these 15 genes between mature pollen and young seedlings indicated that they have distinctive expression patterns. Interestingly, most of these genes are relatively more abundant in vegetative tissue than in pollen, except *AGO5 *and *AGO9 *(see Additional file 3). *AGO5 *is the only member of the plant-specific *MEL1 *subfamily in Arabidopsis. *MEL1 *is required for reproduction in rice [23], however, *AGO5 *knock-out has no obvious phenotype in Arabidopsis [24], indicating functional redundancy among *AGO *family members in mature pollen.

### MicroRNA array revealed that most known miRNAs are down-regulated in mature pollen

**Figure 2:**
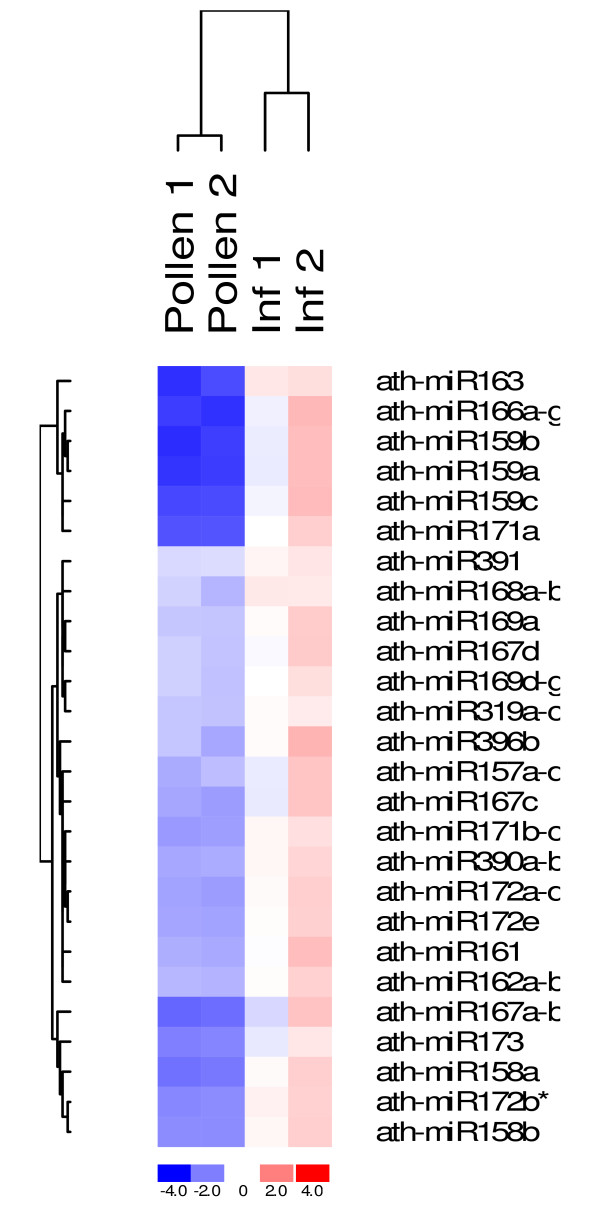
**Heat map and unsupervised hierarchical clustering of 26 differentially expressed miRNAs**. The heat map diagram shows the result of the two-way hierarchical clustering of genes and samples. Each row represents a miRNA and each column represents a sample. The miRNA clustering tree is shown on the left, and the sample clustering tree appears at the top. The colour scale shown at the bottom illustrates the relative expression level of a miRNA across all samples: red represents an expression level above mean, blue represents expression lower than the mean. The clustering is performed on log_2_(Hy3/Hy5) ratio which passed the filtering criteria on variation across samples; standard deviation > 0.50. miR172b* was not followed up in other experiments.

The expression of RNA silencing pathway genes in mature pollen indicates that this pathway may play a role in regulating gene expression during pollen development and function. Studies have indicated that transcripts required for mature pollen functions are produced and stored in pollen, and it is post-transcriptional processes, such as translation, that controls the expression of the functional products [17]. Because of the roles of miRNAs in post-transcriptional gene regulation, it is plausible to assume that miRNAs have important roles in pollen function. To address this question, we decided to examine the expression of all known miRNAs in Arabidopsis mature pollen using microarray technology. The Exiqon miRCURY™ LNA array Version 8.1 [25] was used in this study. The array contains probes for 70 miRNAs from Arabidopsis, representing 121 miRNAs IDs represented in the miRBase . We wanted to examine the miRNAs that are expressed in mature pollen, and also compare the expression level in mature pollen to that in young inflorescence that contains the inflorescence meristem and unopened flower buds from stage 1 to 12 [26]. Total RNA was isolated from these two tissue types from pooled samples, and the RNA samples were delivered to Exiqon where RNA quality control and the array experiment were performed. There were total four RNA samples representing two independent RNA preparations for each tissue that was harvested from different batched of plants. RNAs from all samples were pooled together as the control which was labelled with Hy5 fluorescence dye, and each individual sample was labelled with Hy3. Each array was hybridized with the control sample and an individual sample, and the signal intensity from both channels was analyzed to identify expressed miRNAs and compare their expression level. Among 70 Arabidopsis probes presented on the array, 37 had detectable expression in mature pollen, among which 26 probes have shown significant difference in expression in the two tissue types (Figure 2). Interestingly, most of the expressed miRNAs were less abundant in pollen than in young inflorescence, and only a few of them showed roughly the same expression level in both tissues. We speculate that the reason we could not identify a miRNA that is up-regulated in mature pollen could be due to the fact that all known miRNAs were identified and confirmed in sporophytic tissues. It is possible that we have not found miRNAs that are specific or abundant in mature pollen.

To validate the miRCURY array data, we compared our results with the inflorescence expression data stored in ASRP database [27]. The data in ASRP database was obtained from cDNA library made with RNAs from inflorescence with stage 1 to 12 flowers, which is comparable to our inflorescence sample. Although we can't directly draw a linear correlation between these two sets of data due to the difference in detection methods, we can validate the array experiment based on the presence/absence of expression data. Fifty-six out of the 70 probes on the array have expression data in the ASRP database, and 9 of them were not detected in inflorescence samples by the array experiment, which consisted of more than 12% of the miRNAs represented by the array (Table 1). Since all of the 9 miRNAs were low in abundance, we think that the miRCURY array may not be sensitive enough to detect their expression.

**Table 1:** Comparison of miRNA expression using MiRCURY array and TaqMan miRNA assay

	MiRCURY Array^£^				Taqman MiRNA Assay^¥^					
				
	log_2_(sample/pool)				MP C_T_	Inf C_T_		AS	Slotkin et. al^€^	
						
Name	Inf 1	Inf 2	MP1	MP2	mean	mean	ΔΔC_T_	RP^§^	Inf	MP
156a-f	-0.44	0.27	0.23	0.19	29.85	29.77	-0.9	2	2591	51961

156g	-0.33	0.34	0.16	0.09	35.08	34	0.09*	0	131	678

**156h**					35.49	31.6	2.89	0	1525	611

157a-d	-0.32	0.92	-1.35	-1.04	34.42	31.18	2.26	1	1792	671

158a	0.07	0.77	-2.21	-2.12	32.87	27.9	3.95	8	16305	2307

158b	0.15	0.79	-1.82	-1.82	32.89	30.5	1.39	0	13757	2037

159a	-0.33	1.03	-3.17	-3.04	NA	25.2		67	197957	5540

159b	-0.31	1.04	-3.31	-3.01	NA	24.3		10	189644	5319

159c	-0.17	1.07	-2.87	-2.84	35.85	29.7	4.49	0	35850	1247

160a-c	-0.04	0.39	-0.31	-0.39	30.71	28.95	0.74	17	833	351

161	-0.03	1.04	-1.29	-1.36	33.65	29.7	2.91	71	8245	4737

162a,b	0.05	0.73	-1.13	-1.2	35.35	28.9	5.82	7	123	35

163	0.39	0.51	-3.27	-2.83	36.98	30.5	5.29	3	5665	126

164a,b	NA	NA	NA	NA	36.39	25.6	8.53	21	10771	382

164c	NA	NA	NA	NA	37.04	26.1	10.03	1	10005	349

**165a,b**	0.31	0.58	0	0.06				1	1	15

166a-g	-0.24	1.11	-3.04	-3.25	31.93	24.9	6.4	6	472	33

167a,b	-0.64	0.96	-2.4	-2.29	35.95	25.6	9.48	218	33681	1154

167c	-0.34	0.94	-1.41	-1.55	NA	26.1		1	250	14

167d	-0.09	0.85	-0.76	-0.96	NA	34		4	852	19

168a,b	0.37	0.36	-0.7	-1.18	32.49	28.6	3.56	16	19462	5038

169a	0.07	0.83	-0.91	-0.94	NA	33.8		1	399	7

169b,c	NA	NA	NA	NA	NA	32.5		0	380	7

169d-g	-0.01	0.52	-0.74	-0.98	NA	30.01		2	234	9

170	NA	NA	NA	NA	32.9	29.3	2.95	7	5207	137

171a	0	0.79	-2.7	-2.67	37.0	26.5	9.81	247	39570	922

171b,c	0.14	0.51	-1.6	-1.52	36.28	26.8	8.40	5	4149	91

172a,b	0.08	0.79	-1.47	-1.55	32.69	28.9	3.14	73	8220	8147

172c,d	NA	0.26	-0.36	-0.33	32.47	28.4	3.47	1	7576	8353

172e	0.04	0.75	-1.42	-1.48	32.81	28.9	3.17	6	5245	2320

173	-0.38	0.39	-1.99	-1.93	34.93	29.6	4.71	1	625	501

319a,b	0.07	0.33	-0.93	-0.97	36.18	27.44	8.26	5	14514	713

**319c**					36.97	30.11	6.38	2	13795	653

390a,b	0.14	0.66	-1.39	-1.33	33.15	24.86	7.79	2	8743	695

**391**	0.15	0.42	-0.6	-0.54				3	14	19

394a,b	NA	0.42	-0.93	-0.96	35.84	28.97	6.43	1	21	1

395a,d,e	-0.04	0.11	-0.49	-0.47	NA	34.91		0	139	2

395b,c,f	-0.09	0.22	0.01	-0.07	NA	31.94		0	137	2

396a	NA	0.72	NA	-0.76	34.87	29.34	4.91	6	352	19

396b	0.06	1.18	-0.92	-1.39	NA	27.5		4	371	21

399b,c	-0.21	0.09	-0.31	NA	35.84	32.12	2.91	0	485	106

403	NA	NA	NA	NA	36.61	30.9	4.92	1	696	275

**414**	0.18	0.11	-0.42	-0.66				NA	0	0

419	0.17	0.31	-0.47	-0.32	NA	NA		NA	0	0

**447a,b**	-0.13	0.11	-0.04	-0.15				0	286	49

447c	-0.26	-0.1	0.22	0.23	NA	NA		0	0	0

All miRNA names have been abbreviated. If a probe represents a miRNA family with more than three members, the name is shortened to save space. For example, miR156a-f represents miR156a, b, c, d, e, f. MiRNAs that were not present either on the array or the Taqman assay were in bold. ^£^, the MiRCURY array data were shown as Log_2_(sample/pool). ^¥^, The C_T _value represents means from all the replicates. T-test was performed to evaluate the ΔΔC_T _value for difference that is statically significant (with p < 0.05). The only one that did not pass the test was marked by an asterisk. ^§^, the value in the table represents the normalized read (reads/million) for each miRNA in inflorescence (Col-0) as stored in ASRP database. ^€^, the value represents the un-normalized sequencing reads from the small RNA libraries published by Slotkin et. al. [29]. The whole inflorescence library has 4,158,848 sequences, while the mature pollen library has 1,034,665 sequences. The table only listed miRNAs that were found in at least one of the sources. Inf, inflorescence; MP, mature pollen. NA, the miRNA was either not detected or unavailable.

**Figure 3:**
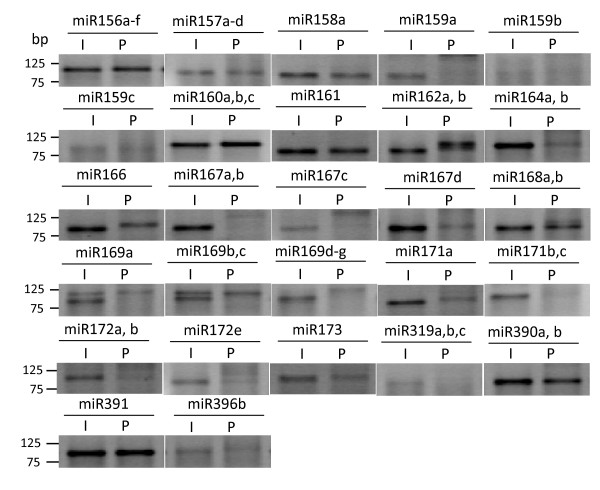
**Expression of 27 miRNAs in inflorescence and mature pollen by RT-PCR**. The adaptor sequence is 46 bp long. Depending on the length of the miRNA and the number of nucleotides added during the polyadenylation step, the PCR products range from 70 to 90 bp. I, inflorescence; P, mature pollen.

To further confirm our findings, we examined the expression of 27 miRNAs by using RT-PCR technique. The 27 miRNAs included 24 miRNAs that were shown to be down-regulated in mature pollen, 2 that had shown no significant difference in expression level in two different tissue types (miR156a,b,c,d,e,f and miR160a,b,c), and 2 that were not detected by the array in inflorescence samples but have expression data in ASRP database (miR164a,b, miR396). Based on the RT-PCR analyses, 5 miRNAs were expressed in inflorescence but not detectable in mature pollen (miR159a; miR167a,b; miR167c; miR169d,e,f,g; miR171b,c), 3 of the miRNAs (miR159b; miR159c; miR319a,b,c) were barely detectable in both tissue types. The expression of the remaining miRNAs was detected in pollen and inflorescence. Among them, 7 had roughly the same expression level in both tissue types (miR156a,b,c,d,e,f; miR160a,b,c; miR161; miR162a,b; miR390a,b; miR391), while the others were expressed at much lower levels in mature pollen (Figure 3). Since RT-PCR is not really quantitative, we can't conclude whether the difference observed in two tissue types matched what we have obtained from the miRCURY array. However, the discrepancies observed from the two approaches raise our concerns regarding the accuracy, sensitivity and reproducibility of the array experiment. For instance, the array failed to detect the expression of miR164a,b and miR396, whose expression in inflorescence was confirmed by both ASRP database and RT-PCR. Several miRNAs (miR159a; miR159b; miR167a,b; miR167c; miR169d,e,f,g) were not detected in mature pollen by RT-PCR, however, they were considered to be expressed based on the array results.

Overall, the miRNA expression profiling by the miRCURY array has provided some valuable information. However, the additional efforts required to validate the results make this approach less attractive for quantifying the expression of miRNAs. The microarray experiment used in a transcriptome study can easily justify its cost and the sensitivity issues because it is designed for thousands of genes. However, microarray for miRNAs may not be so worthwhile, considering the number of genes analyzed, the cost, and the outcome.

### Quantitative analysis of miRNA expression using TaqMan assay

The array experiment revealed several important facts regarding miRNA expressed in mature pollen, however, the results were not very satisfactory. Data from two inflorescence samples had shown large variation (Figure 2), and the high cost of the experiment has limited us to include more biological replicates in the experiment. In addition, microarray experiments in general are not as sensitive in detecting low abundant genes compared to PCR based assay, and it tends to generate false positive or false negative results. To complete this study, we decided to examine the expression level of miRNAs in our samples by using real time-PCR based on TaqMan MicroRNA assay (Applied Biosystems, Foster City, CA). TaqMan miRNA assay is based on stem-loop RT-PCR detection method, and the TaqMan probe in each assay was designed for a specific miRNA. The assay has been tested to be specific for mature miRNA [28]. Using assays for 65 miRNAs, we confirmed the expression of 22 miRNAs in mature pollen, and identified 8 additional miRNAs (miR156h; miR164a,b; miR164c; miR170; miR319c; miR396a, miR399b,c; miR403) that were expressed at low levels in mature pollen. However, the expression of 11 miRNAs that were detected in mature pollen on the array was not confirmed by the Taqman miRNA assays (Table 1). We also cross examined the Taqman assay results with the results from ASRP database and RT-PCR experiment (Figure 3 and Table 1) to validate the assay. The Taqman assay was successful in detecting the expression of all miRNAs with ASRP inflorescence expression data. In addition, the assay identified 8 additional miRNAs that were low abundant in inflorescence sample. When compared with the RT-PCR results, expression patterns of most miRNAs examined using these two approaches were for the most part consistent with the exception of miR169a and miR169b, c. These two miRNAs were detected by RT-PCR, however, Taqman assay did not detect their expression in mature pollen. Further analysis of the gel picture indicated that there were two products amplified with the primers for miR169a and miR169b, c. Only the larger size product was detected in mature pollen. Since the primers used in RT-PCR reactions only differed by one base pair, we speculated that the larger product was due to non-specific amplification.

Slotkin et. al. have recently sequenced small RNA libraries made from whole inflorescence and mature pollen [29]. To validate the expression profile generated in this study, we also compared our analyses with their sequencing data . All the miRNAs detected by real-time PCR have been found in the sequencing database. However, there were nine miRNAs (miR159a; miR159b; miR167c; miR167d; miR169a; miR169d,e,f,g; miR395a,d,e; miR395b,c,f; miR396b) that were found in the sequencing data, but not detected by real-time PCR (Table 1). Seven out of these 9 miRNAs had very low sequencing frequency, and their expression cannot be confidently confirmed until the sequencing data has been normalized. However, the other two miRNAs (miR159a and miR159b) have been sequenced more than 5,000 times in mature pollen. Since the expression of these two miRNAs was not detected by either real-time PCR or regular RT-PCR (Table 1 and Figure 3), further analysis of the sequencing data would be required to solve the discrepancy. We also cannot directly compare the difference in expression level between inflorescence and pollen samples in our analyses with that based on the sequenced libraries. Because the small RNA library for inflorescence was made with whole inflorescence that included open flowers, whereas our inflorescence sample only included stage 1–12 flowers. In addition, these sequencing reads have not been normalized, which prevented us from comparing the relative abundance of each miRNA between two samples. Nevertheless, the sequencing results have suggested that most of the miRNAs are expressed at lower level in mature pollen, which is consistent with our findings.

### MicroRNA expression was correlated with their target gene expression in pollen

Since we were able to detect the expression of RNA silencing pathway genes and some miRNAs in mature pollen, we wanted to know whether these miRNAs regulate the expression of their target genes. We have chosen three miRNA families (miR156a,b,c,d,e,f; miR160a,b,c; miR161) that are relatively abundant in mature pollen based on our analyses. We first identified the target genes of these miRNAs on ASRP database , then analyzed their expression in immature male gametophyte, mature pollen and sperm cells based on the available transcriptome data [17,18,30]. If these miRNAs indeed are functional, we expect to see reduced expression of their target genes in the corresponding tissue. By comparing the target gene expression in different stages of male gametophyte and sperm cells, we indeed found an anti-correlation in the expression of each miRNA and its target genes in most of the cases (see Additional file 4). The only two exceptions were: *At1g53160 *(*SPL4*), a target of miR156 family, and *At3g16710*, a target of miR161. The expression of *SPL4 *in mature pollen was not consistent based on the two transcriptome studies [17,18], therefore, we have ruled it out from the analysis. *At3g16710 *is one of the 441pentatricopeptide (PPR) repeat-containing proteins found in Arabidopsis that are important for RNA processing and translation inside organelles [31]. The transcriptome data indicated that *At3g16710 *was only present in mature pollen grains. We speculate that this gene could be sperm specific, therefore, its expression may not be affected as much by miRNAs located in the vegetative cells.

In addition to our analyses based on transcriptome data, a recent study by Grant-Downton et. al. [32] has confirmed the function of miRNAs in mature pollen by identifying the cleavage products generated from targets of miR160 and miR172. Based on these experiments, we concluded that miRNAs have important regulatory roles in controlling gene expression in mature pollen.

## Conclusion

Using miRCURY LNA array technology and TaqMan miRNA assays, we have identified a total 45 miRNAs that are expressed in Arabidopsis mature pollen, among which 22 have been confirmed with both technologies. Interestingly, most of the miRNAs are very low abundant in mature pollen, with just a few exceptions. Based on the real-time PCR results, the expression of miR156g was about the same in these two tissue types, and the expression of miR160a, b, c in pollen was less than two-fold lower than that in inflorescence. The only miRNA that has higher expression in pollen was miR156a, b, c, d, e, f. Our analyses of the transcriptome data for some miRNA target genes and the results from Grant-Downton et. al. [32] have supported the function of miRNAs in mature pollen. However, genetic study based on mutants carrying mutations in genes in the RNA silencing pathway or MIR genes have revealed very little about their function in mature pollen. This could be explained by functional redundancy or the difficulty in isolating gametophytic mutants. In summary, this study has used different technologies to examine miRNA expression in mature pollen, and generated valuable data that can be used to evaluate the roles of miRNAs in pollen function.

The direct comparison of the two techniques commonly used in quantifying miRNA expression suggests that users should take precaution when using microarray technology to examine miRNA expression, since the experimental cost may not be very well justified by the outcome. The microarray technology is high throughput and suitable for expression profiling of thousands of genes. However, using array to analyze miRNA expression may not be cost-effective because the number of miRNA genes needed to be analyzed is not very high and the technology still has to deal with the inconsistencies for low abundant miRNAs.

## Methods

### Plant materials

Arabidopsis plants (Col-0) were grown in the growth chamber at 22°C with long day conditions (16 hr light/8 hr dark). Mature pollen was harvested using a modified hand-held vacuum [20], and young inflorescence was harvested directly from the plant. Plant materials were quickly frozen in liquid nitrogen once harvested, and stored in -80°C freezer until the next step.

### RNA preparation

Total RNA was isolated from each sample using the TRIzol reagent (Invitrogen, Carlsbad, CA) following the manufacturer's instruction. RNA samples for the array experiment were cleaned using the RNeasy kit (Qiagen, Hamburg, Germany) with slight modification to preserve miRNAs. Basically, 350 μl Buffer RLT and 3.5 volume of 100% ethanol were added to 50 μl of RNA sample, and the mixture was added onto an RNeasy Mini spin column. After centrifugation, the column was washed twice with 500 μl buffer RPE, and the RNA was eluted with 30 μl RNase-free water. Samples were concentrated to at least 1 μg/μl, and delivered to Exiqon on dry ice for the miRCURY array experiment. Exiqon performed the array experiments and analyzed the data. For TaqMan miRNA assay, RNA samples were cleaned with TURBO DNase (Applied Biosystems, Foster City, CA).

### Reverse Transcriptase-PCR

For the RT-PCR experiment, 1 μg of total RNA from each sample was converted to cDNA in a 20 μl reaction containing 1 μl Supercript II reverse transcriptase (Invitrogen, Carlsbad, CA), 1 μl RNasin (Promega, Madison, WI), 2 μl DTT (100 mM), 1 μl Oligo dT primer (20 μM), and 4 μl 5× reaction buffer. One microliter of cDNA sample was used in subsequent PCR reactions using gene-specific primers with the following cycle conditions: 94°C, 30 second; 57°C, 30 second; 72°C, 1 minute for 30 cycles. See Additional file 5 for all primers used in the experiment.

RT-PCR for miRNAs was performed using QuantiMir RT kit (System Biosciences, Mountain View, CA) following the manufacturer's instruction. Briefly, 1 μg of RNA from each sample was polyadenylated, and then converted to cDNAs with a unique adaptor in the presence of reverse transcriptase, and the cDNAs were amplified with specific miRNA primer in combination with the universal adaptor to examine the expression of a particular miRNA. Primers and product sizes were listed in Additional file 5. Some of the PCR products were cloned and sequenced to confirm that a specific miRNA was amplified.

### Taqman MiRNA Assay

To make cDNA for each Taqman miRNA assay, 5 ng or 10 ng of total RNA was incubated with 0.15 μl dNTPs (100 mM), 1.5 ul 10× reaction buffer, 0.19 μl RNase inhibitor, 1 μl Reverse transcriptase, and 3 μl gene-specific primer in a 15-μl reaction. The real-time PCR for each assay was set up as a 20 μl reaction including 10 μl Taqman 2× Universal PCR master mix, 1 μl 20× Taqman Assays that includes gene-specific primers and Taqman probe, and 1.5 μl of cDNA. 5S rRNA was used as the endogenous control for comparative C_T _analyses. Primers and Taqman probe for the 5S rRNA were designed using Primer Express Software (Version 3.0) (Applied Biosystems, Foster City, CA). 5S rRNA Forward: 5'-CGATGAAGAACG TAGCGAAATG-3'; 5S rRNA Reverse: 5'-CTCGATGGTTCACGGGATTC-3'; Taqman Probe: 5'-TACTTGGTGTGAATTGC-3'. TURBO DNase-treated RNA samples were converted to cDNA using High-capacity cDNA reverse transcriptase kit (Applied Biosystems, Foster City, CA) for amplifying 5S rRNA. A standard curve was used to check the efficiency of the primers and probe. Taqman assay for 5S RNA was set up as a 20 μl reaction containing 10 μl Taqman 2× Universal PCR master mix (Applied Biosystems, Foster City, CA), 1 μl of each primers (900 nM), 1 μl Taqman probe (250 nM), and 1.5 μl cDNA sample. All real-time PCR reactions were performed in a StepOne real-time PCR machine (Applied Biosystems, Foster City, CA) with following cycling conditions: 95°C for 10 minutes to activate the enzyme; then repeat 95°C for 15 seconds and 60°C for 1 minute for 40 cycles.

### Real-time PCR reaction setup and data analyses

There were three RNA samples for each tissue type. Two of the mature pollen samples were the ones used in the miRCURY array, while the third one was isolated from a different batch of plants. For inflorescence samples, one was the Inf2 sample used in miRCURY array, and the other two samples were prepared from new plant materials grown under the same conditions. For Taqman miRNA assay, each RNA sample was reverse transcribed as described above and the assay for each miRNA target was set up in triplicate reactions. Each 48-well reaction plate contained reactions for the endogenous control (5S rRNA) and an individual miRNA target for all six biological samples. Non-template controls were also set up as triplicates. Results were exported to calculate mean C_T_, which was then used to calculate ΔC_T _value for each miRNA target based on the formula: ΔC_T _= C_T_(target miRNA) - C_T_(5S rRNA). ΔΔC_T _for each miRNA target was calculated using the formula ΔΔC_T _= ΔC_T _(pollen) -ΔC_T _(inflorescence). ΔC_T _(pollen) and ΔC_T _(inflorescence) for each miRNA target were used to run a two-sample t-test with Prism (v. 5.0, GraphPad Software, Inc., La Jolla, CA) to detect statistically significant difference in expression between pollen and inflorescence samples. The ones with p < 0.05 were considered as statistically significant.

## Abbreviations

RISC: RNA-induced silencing complexes; AGO: Argonaute; PMC: pollen mother cells; MP: mature pollen; DCL: Dicer-like; RDR: RNA-dependent RNA polymerases; DRB: Double-stranded RNA binding protein; FACS: Fluorescence-Activated Cell Sorting; Inf: inflorescence; RT-PCR: reverse transcriptase-PCR.

## Authors' contributions

CC took care of the plants and isolated total RNA. BS designed and performed other experiments. BS wrote and edited the manuscript. All authors read and approved the final manuscript.

## Supplementary Material

Additional file 1 **Expression of *ACTIN7 *in inflorescence and mature pollen by RT-PCR**. The amplification from cDNA was indicated by the arrowhead. M, DNA standard; I, inflorescence; P, mature pollen. Click here for file

Additional file 2 **Expression of RNA silencing pathway genes in Arabidopsis mature pollen**. ^1^Both microarray studies were done using the Affymetrix ATH1 Genome Array. ^2^Data from Honys & Twell [17] were normalized. ^3^Data from Pina et.al. [18] are represented in two columns: the left column was the raw signal intensity, the right column was the present (P)/absent (A) call after data normalization. ^4^RT-PCR results were represented as follow: ++, strongly expressed; +, expressed; -, non-detectable. ^5^Data for *AGO8 *was not reported in Pina et. al. [18]. ^6^*RDR3 *and *RDR4 *are not represented on the ATH1 chip. Click here for file

Additional file 3 **Expression of RNA silencing pathway genes in Arabidopsis 12-day-old seedlings by RT-PCR**. *ACT7*(*ACTIN7*) was used as a positive control to ensure the quality of RNA and cDNA. PCR products amplified from cDNA are indicated by asterisks. PCR products at higher molecular weight in each sample were amplified from genomic DNA. *DCL: Dicer-like; AGO, Argonaut; RDR, RNA-dependent RNA polymerases; DRB, Double-stranded RNA binding protein*. Click here for file

Additional file 4 **Target gene expression in young male gametophyte, mature pollen and sperm cells**. All microarray studies were done using the Affymetrix ATH1 Genome Array. ^1^Data from Honys & Twell [17] were normalized. ^2&3 ^Data from Pina et.al. [18] and Borges et. al. [30] were represented in two columns: the left column was the raw signal intensity, the right column was the present (P)/absent (A) call after data normalization. NA, the expression of the gene was not available. The table only included target genes that have expression data in at least one sample. UNM, uninucleate microspore; BCP, bi-cellular microspore; TCP, tri-cellular microspore; MP, mature pollen. Click here for file

Additional file 5 **Primers used in RT-PCR experiments.** Click here for file
