# Supplementary figures and images for: Profiling microRNA expression in Arabidopsis pollen using microRNA array and real-time PCR
Source: BMC Plant Biol. 2009 Jul 10;9:87. doi: 10.1186/1471-2229-9-87 (PMC2715406; doi:10.1186/1471-2229-9-87)

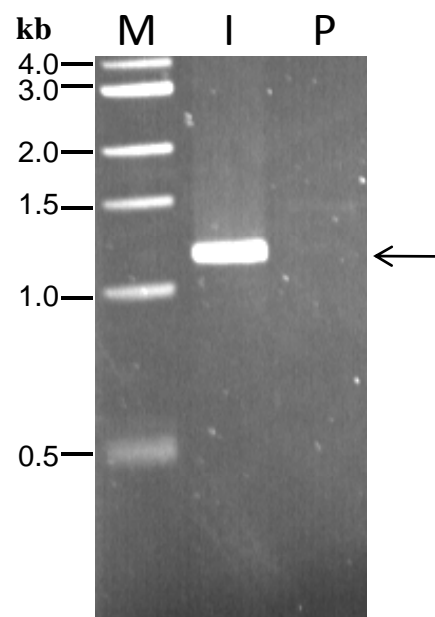

Supplement: Additional file 1 — Expression of ACTIN7 in inflorescence and mature pollen by RT-PCR. The amplification from cDNA was indicated by the arrowhead. M, DNA standard; I, inflorescence; P, mature pollen. [file 1471-2229-9-87-S1.pdf]

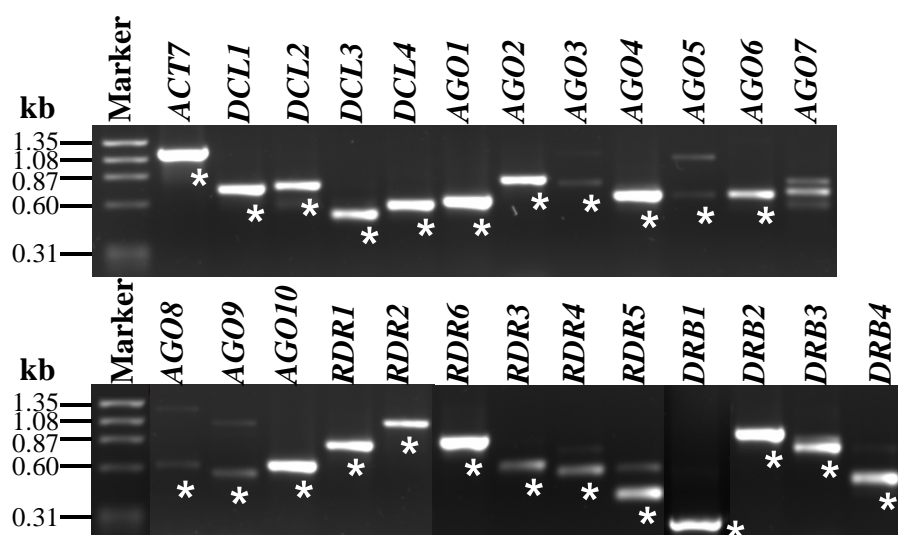

Supplement: Additional file 3 — Expression of RNA silencing pathway genes in Arabidopsis 12-day-old seedlings by RT-PCR. ACT7(ACTIN7) was used as a positive control to ensure the quality of RNA and cDNA. PCR products amplified from cDNA are indicated by asterisks. PCR products at higher molecular weight in each sample were amplified from genomic DNA. DCL: Dicer-like; AGO, Argonaut; RDR, RNA-dependent RNA polymerases; DRB, Double-stranded RNA binding protein. [file 1471-2229-9-87-S3.pdf]
