# Supplementary material for: Profiling microRNA expression in Arabidopsis pollen using microRNA array and real-time PCR
Source: BMC Plant Biol. 2009 Jul 10;9:87. doi: 10.1186/1471-2229-9-87 (PMC2715406; doi:10.1186/1471-2229-9-87)
Supplement: Additional file 2 — Expression of RNA silencing pathway genes in Arabidopsis mature pollen. 1Both microarray studies were done using the Affymetrix ATH1 Genome Array. 2Data from Honys & Twell [17] were normalized. 3Data from Pina et.al. [18] are represented in two columns: the left column was the raw signal intensity, the right column was the present (P)/absent (A) call after data normalization. 4RT-PCR results were represented as follow: ++, strongly expressed; +, expressed; -, non-detectable. 5Data for AGO8 was not reported in Pina et. al. [18]. 6RDR3 and RDR4 are not represented on the ATH1 chip. [file 1471-2229-9-87-S2.pdf]

Gene Name	Gene ID	Microarray Data[1]			RT-PCR[4]
		Honys & Twell[2]	Pina et. al.[3]		
DCL1	At1g01040	0	58.4	A	+ +
DCL2	At3g03300	0	29.3	A	+
DCL3	At3g43920	0	50.9	A	+
DCL4	At5g20320	0	87.1	A	+
AGO1	At1g48410	0	33.5	A	+
AGO2	At1g31280	0	60.0	A	+
AGO3	At1g31290	261.739	46.2	P	-
AGO4	At2g27040	0	69.4	A	+
AGO5	At2g27880	0	184.1	A	++
AGO6	At2g32940	154.036	89.7	A	+
AGO7(ZIPPY)	At1g69440	0	68.0	A	+
AGO8	At5g21030	0	Not in the data[5]		+
AGO9	At5g21150	293.4005	200.7	A	++
AGO10(PIN/ZWL)	At5g43810	0	45.0	A	+
RDR1	At1g14790	0	53.2	A	-
RDR2	At4g11130	0	29.2	A	+
RDR3	At2g19910	Not on the chip[6]			++
RDR4	At2g19920	Not on the chip[6]			++
RDR5	At2g19930	0	81.0	A	+
RDR6/SDE1	At3g49500	0	273.0	A	+
DRB1/Hyl1	At1g09700	0	135.6	A	+
DRB2	At2g28380	0	82.0	A	-
DRB3	At3g26932	0	69.4	A	++
DRB4	At3g62800	369.2595	269.4	P	++
