# Supplementary material for: Profiling microRNA expression in Arabidopsis pollen using microRNA array and real-time PCR
Source: BMC Plant Biol. 2009 Jul 10;9:87. doi: 10.1186/1471-2229-9-87 (PMC2715406; doi:10.1186/1471-2229-9-87)
Supplement: Additional file 5 — Primers used in RT-PCR experiments. [file 1471-2229-9-87-S5.pdf]

Primer name	Primer sequence (5'-3')	Product size (bp)	
		genomic DNA	cDNA
ACT1-F	TGCACTTCCACATGCTATCC	717	606
ACT1-F	TGAACAATCGATGGACCTGA		
ACT7-F	AAAATGGCCGATGGTGAGG	1450	1172
ACT7-R	ACTCACCACCACGAACCAG		
AGO1-F	GTTGTGGCATCTCAGGATTG	1143	601
AGO1-R	AACTGAGCGTGTGCATCTTG		
AGO2-F	CGGTTCAGTAGGAGGAGTCG	990	781
AGO2-R	GAACGCCAACACCGAGTAAT		
AGO3-F	GGGCTCGTAAGCCTCAACTT	1102	741
AGO3-R	ACGCCAACAAAGAGGAGTTC		
AGO4-F	CTGTTCGGACACAGCCTTCT	1231	618
AGO4-R	CACCATGGCTTGATGATGTC		
AGO5-F	ACAGCCTGGAGAGGACTCAA	1015	607
AGO5-R	GCAGAGGTTGTTTGTGAGCA		
AGO6-F	CGCTCTTCCAACCTATCGAG	1276	638
AGO6-R	TTCGACATTCTCGTGCAGAC		
AGO7-F	TCTTCCTCCTCCTCCTCCTC	1041	644
AGO7-R	CCCAATCCTCTCCTTCCTTT		
AGO8-F	GTGCGTACACAGTCGCCTAA	1172	649
AGO8-R	CCGCCAAATGAGCATAACAT		
AGO9-F	GGATGGTGTAAGCGAGTCTC	972	490
AGO9-R	CTCCAGGTGTGGTGATTCCT		
AGO10-F	GATCCTGTTCGCGGTACTGT	1075	550
AGO10-R	AGAAACGTGCTCGAAATGCT		
DCL1-F	TTCGGGAATTTGTGAAGGAG	901	718
DCL1-R	AGAAGAACCGCAGCTGAATC		
DCL2-F	TGTTCATGAATTGGGTTGGA	920	743
DCL2-R	ATCAGAGGCCTTTGCTGTGT		
DCL3-F	GATGGTGTCAAAGCACAAGC	754	571
DCL3-R	CTTGTCAGGCCTTGCTTCTC		
DCL4-F	GGGAAATATCAGCGACGAAA	906	582
DCL4-R	ATATTGGGCGCATCTTCAAC		
DRB1-F	CTCTCCGGGAATTAGCAAAA	661	258
DRB1-R	CAGTTCTCCCAGCGCTAATC		
DRB2-F	GCTGCTTGGTCTTCCCTAAA	960	844
DRB2-R	GTCGCTCTCTTGCTGTCTCC		
DRB3-F	ACCTGGTCACATCCCAACAT	779	700
DRB3-R	CCTTGTGATGAACGGTGATG		
DRB4-F	AGCGCTGCTAAAGTTGCATT	705	447
DRB4-R	ATTCTGAGGCATCCACGAAG		
RDR1-F	TATGGTGGACTGCGTTGTGT	912	735

RDR1-R	GCTCTTCCAATCGATTCTGC		
RDR2-F	TGAGGTCCATTTCGAAGAAAA	1178	975
RDR2-R	TCAACGCTCTGTCTCTGTGC		
RDR3-F	ATAGGGATGGCTGCAGATTG	814	590
RDR3-R	TTCGCAACACCTTCCTGAAT		
RDR4-F	GTGAACCGTGGGTGAGTTCT	711	555
RDR4-R	TCATCGCTTGACACATCTCC		
RDR5-F	ACTCCCGTGTTTCGAAGATG	539	364
RDR5-R	CACAGATGGTGCACACACTAAA		
RDR6-F	GAAGAGTTTAGGCCGTGCT	1259	837
RDR6-R	CGTGACCGGATTTTGATTCT		
miR156a-f	TGACAGAAGAGAGTGAGCAC		
miR157a-d	TTGACAGAAGATAGAGAGCAC		
miR158a	TCCCAAATGTAGACAAAGCA		
miR159a	TTTGGATTGAAGGGAGCTCTA		
miR159b	TTTGGATTGAAGGGAGCTCTT		
miR159c	TTTGGATTGAAGGGAGCTCCT		
miR160a,b,c	TGCCTGGCTCCCTGTATGCCA		
miR161	TGAAAGTGACTACATCGGGGT		
miR162a,b	TCGATAAACCTCTGCATCCAG		
miR164a,b	TGGAGAAGCAGGGCACGTGCA		
miR166	TCGGACCAGGCTTCATTCCCC		
miR167a,b	TGAAGCTGCCAGCATGATCTA		
miR167c	TAAGCTGCCAGCATGATCTTG		
miR167d	TGAAGCTGCCAGCATGATCTGG		
miR168a,b	TCGCTTGGTGCAGGTCGGGAA		
miR169a	CAGCCAAGGATGACTTGCCGA		
miR169b,c	CAGCCAAGGATGACTTGCCGG		
miR169d-g	TGAGCCAAGGATGACTTGCCG		
miR171a	TGATTGAGCCGCGCCAATATC		
miR171b,c	TTGAGCCGTGCCAATATCACG		
miR172a,b	AGAATCTTGATGATGCTGCAT		
miR172e	GGAATCTTGATGATGCTGCAT		
miR173	TTCGCTTGCAGAGAGAAATCAC		
miR319a,b,c	TTGGACTGAAGGGAGCTCCCT		
miR390a,b	AAGCTCAGGAGGGATAGCGCC		
miR391	TTCGCAGGAGAGATAGCGCCA		
miR396b	TTCCACAGCTTTCTTGAACTT		
